# Transgenic high-lysine rice – a realistic solution to malnutrition?

**DOI:** 10.1093/jxb/erw254

**Published:** 2016-07-07

**Authors:** Wenyi Wang, Gad Galili

**Affiliations:** Department of Plant Science, Weizmann Institute of Science, Rehovot 76100, Israel

**Keywords:** Aspartate kinase (AK), dihydrodipicolinate synthase (DHPS), lysine, lysine ketoglutaric acid reductase/saccharopine dehydropine dehydrogenase (LKR/SDH), rice (*Oryza sativa*).

**Lysine is considered an ‘essential amino acid’ required at sufficient levels to prevent malnutrition and serious diseases, particularly in developing countries. It is mostly obtained from various plant foods. In the current issue (pages 4285–4296) Yang and colleagues report on successful stimulation of lysine biosynthesis and suppression of its catabolism in transgenic rice plants without changing the plant phenotype. This approach led to the production of high-lysine rice plants, rendering them as nutritionally favourable crops.**

Lysine is considered the first limiting essential amino acid in cereal and legume crops – i.e. it is present in the smallest quantity (Galili *et al.*, 1994). This restricted content significantly reduces the nutritional values of these crops to 50–70% compared with those containing more balanced levels (Galili and Amir, 2013). As an ‘essential amino acid’, not produced in the bodies of humans and farm animals, lysine must be obtained from other sources. It is quite extensively present in livestock-derived foods, such as meat, eggs and cheese. However, where diets rely on plant-derived foods – which is the case for huge populations living in poverty in developing countries – people suffer from insufficient lysine levels, leading to vulnerability to disease, decreased levels of blood proteins and retarded mental and physical development in young children (Galili and Amir, 2013). To prevent this, enriching the content of lysine in those crop plants which serve as the major sources of human foods and livestock feed in these countries is essential. Among the cereal crops, rice (*Oryza sativa*) is a stable source of calories and protein intake for approximately one-third of the world’s population (Kusano *et al.*, 2015).

Lysine metabolism in plants has been studied for over 50 years, since the discovery of the maize high-lysine mutant *opaque-2* (*o2*), which contains low levels of lysine-poor seed storage proteins (zeins) and consequently an increased lysine and tryptophan content compared with the wild type (Mertz *et al.*, 1964). However, enrichment of lysine levels in crops using classical genetics and breeding approaches is difficult because: (i) lysine synthesis is highly negatively regulated by a feedback inhibition loop in which lysine feedback inhibits the activity of dihydrodipicolinate synthase (DHPS), the first enzyme of the lysine biosynthesis pathway, slowing down its synthesis (Box 1); and (ii) lysine is efficiently degraded by its catabolism into the tricarboxylic (TCA) cycle, a pathway initiated by the bi-functional enzyme LKR/SDH (Box 1), which exhibits both lysine-ketoglutarate reductase (LKR) and saccharopine dehydrogenase (SDH) activities (Karchi *et al.*, 1995). Nevertheless, it is by careful engineering of both of the above enzymes (DHPS and LKR/SDH) that Yang *et al.* (2016) have now developed two pyramid transgenic lines with free lysine content elevated to 25-fold compared to wild type without changing the plant phenotype. This is an important achievement.

## Enhancing lysine levels

A recent study reported the lysine biofortification of seeds of rice by overexpressing genes encoding two endogenous rice histone proteins that are naturally enriched in lysine levels. Expression was achieved using a modified rice glutelin 1 promoter, which is specifically expressed at a relatively low level in developing seeds where the glutelin proteins are synthesized. The choice of a weak seed-specific promoter was intended to prevent physiological problems, such as germination efficiency and vegetative growth, enabling an enhancement of lysine level by a meaningful (up to 35%) amount with no negative impact on the levels of other amino acids. This increase could also meet the nutritional standards of the World Health Organization, offering improved solutions for consumption of human foods and livestock feeds (Wong *et al.*, 2015).

A different approach to that taken by Wong *et al.* (2015) is through metabolic engineering. A number of the earliest of these studies aiming to improve lysine content in plants concentrated on expressing lysine feedback-insensitive DHPS enzymes of lysine biosynthesis under the control of constitutive or seed-specific promoters, so important for cereals where the intention is fortification for human consumption (Karchi *et al.*, 1994; Falco *et al.*, 1995). Expression in soybean and canola seeds led to a notable increase of seed free lysine (Falco *et al.*, 1995). Among these early studies, Keeler *et al.* (1997) also indicated that *de novo* expression of alpha-helical coiled-coil proteins might also increase the accumulation of lysine in mature tobacco seeds.

**Box 1. Biosynthesis and catabolism of lysine in plants**

The pathways leading to and from lysine (biosynthesis/catabolism) also highlight targets for engineering. Enzymes are indicated in purple text. The dashed arrow indicates several non-specified enzymatic reactions. Abbreviations: AK, Asp kinase; DHPS, dihydrodipicolinate synthase; LKR, lysine ketoglutaric acid reductase; SDH, saccharopine dehydropine dehydrogenase; ASD, aminoadipic-semialdehyde dehydrogenase.

**Figure:**
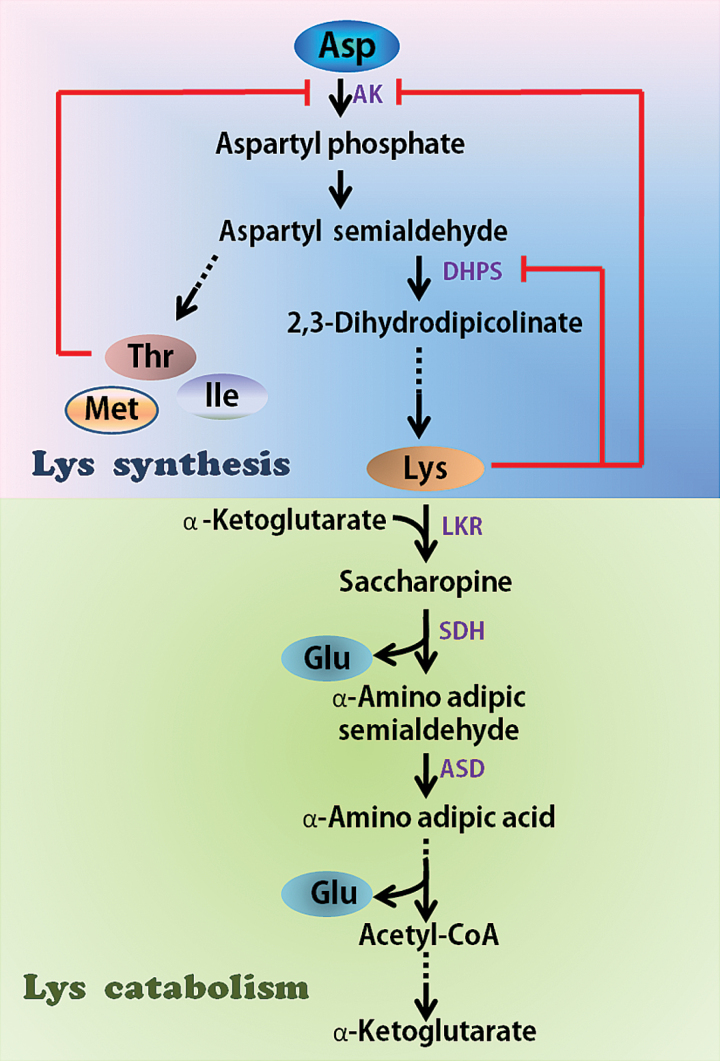

Elevating lysine content by enhancing its synthesis is one way of improving the nutritional quality of crops, but another useful approach is to prevent degradation (catabolism) into the TCA cycle. A third way forward is to combine these in the same crop plant. For example, Zhu and Galili (2004) further expressed the bacterial DHPS (with embryo-specific promoter) in an Arabidopsis knockout mutant lacking LKR/SDH and therefore getting round the problem of lysine catabolism. This approach caused a significant, 64-fold increase in lysine level in the seeds, but was also associated with a prominent reduction of germination rate, indicating that more moderate enrichments of lysine levels would be likely to be more successful. Hence, the balance of synthesis and degradation of lysine is a key regulatory point not only for controlling lysine level, but also improvement of the nutritional quality of crops, and similar findings were also previously reported in maize (Frizzi *et al.*, 2008). Consequently, in subsequent studies, research increasingly focused on the expression of bacterial DHPS enzyme of lysine biosynthesis, specifically in the developing seeds, combined with suppression of lysine catabolism into the TCA cycle by suppressing the activity of either the bi-functional LKR/SDH enzyme of lysine catabolism or the suppression of the LKR enzyme alone. This approach led to significantly increased lysine in various plants (Zhu and Galili, 2003, 2004; Frizzi *et al.*, 2008).

## Getting the balance right

Enhancing the synthesis or reducing the catabolism of lysine can elevate lysine content levels in plants, but lysine overproduction results in increased lysine degradation. Therefore, how to balance the synthesis and catabolism of lysine? It’s the key point of control for lysine levels.

Long *et al.* (2013) showed that the level of LKR/SDH was significantly enhanced in the developing seeds of rice Asp kinase (AK)/DHPS overexpression lines. This means that the effect of transgene AK/DHPS was counterbalanced by the activity of lysine catabolism in sustaining a steady level of lysine. Overexpression of AK and DHPS only increased the free lysine level by 1.1-fold compared with the wild type; however, the LKR-RNAi line showed a 10-fold increase, while combined expression of AK and DHPS and interference of LKR/SDH to achieve both metabolic effects meant a substantial increase in the free lysine content to 60-fold. Similar findings were previously reported in Arabidopsis (Zhu and Galili, 2004) and maize (Frizzi *et al.*, 2008).

Taking this one step further, Yang *et al.* (2016) show a perfect example of meaningful enhancement of lysine levels in transgenic rice by using a combined enhancement of lysine synthesis and suppression of its catabolism. However, in previous studies, the high lysine transgenic plants were typically accompanied by obvious changes of seed phenotype, including oil content, germination and yield (Shaul and Galili, 1993; Tzchori *et al.*, 1996; Zhu and Galili, 2003; Angelovici *et al.*, 2011). In the present study, there was no difference in major agronomic traits, including yield. Furthermore, the promoter used enabled specific accumulation of lysine in the seeds, the main organs consumed in human foods and livestock feeds.

The potential is there for this to solve major problems of malnutrition worldwide, but particularly in developing countries. So far, a maize cultivar, LY038, developed by genetic engineering to have a high lysine content, has been approved for commercial use in a number of countries, including in Africa (Dizigan *et al.*, 2007). The findings of Yang *et al.* (2016) are similarly favourable to commercialization. Meanwhile, there is strong public debate regarding the safety of genetically modified plants and, as with all other GM crops, the opportunities for use of GM rice with enhanced lysine will depend on public acceptance.

## References

[CIT0001] Angelovici R Fait A Fernie AR Galili G 2011 A seed high-lysine trait is negatively associated with the TCA cycle and slows down Arabidopsis seed germination. New Phytologist 189, 148–159. 20946418 10.1111/j.1469-8137.2010.03478.x

[CIT0002] Dizigan MA Kelly RA Voyles DA Luethy MH Malvar TM Malloy KP 2007 High lysine maize compositions and event LY038 maize plants. United States Patent No. 7157281.

[CIT0003] Falco SC Guida T Locke M Mauvais J Sanders C Ward RT Webber P 1995 Transgenic canola and soybean seeds with increased lysine. Bio-Technology 13, 577–582. 9634796 10.1038/nbt0695-577

[CIT0004] Frizzi A Huang S Gilbertson LA Armstrong TA Luethy MH Malvar TM 2008 Modifying lysine biosynthesis and catabolism in corn with a single bifunctional expression/silencing transgene cassette. Plant Biotechnology Journal 6, 13–21. 17725550 10.1111/j.1467-7652.2007.00290.x

[CIT0005] Galili G Amir R 2013 Fortifying plants with the essential amino acids lysine and methionine to improve nutritional quality. Plant Biotechnology Journal 11, 211–222. 23279001 10.1111/pbi.12025

[CIT0006] Galili G Karchi H Shaul O Perl A Cahana A Tzchori IB Zhu XZ Galili S 1994 Production of transgenic plants containing elevated levels of lysine and threonine. Biochemical Society Transactions 22, 921–925. 7698485 10.1042/bst0220921

[CIT0007] Karchi H Shaul O Galili G 1994 Lysine synthesis and catabolism are coordinately regulated during tobacco seed development. Proceedings of the National Academy of Sciences, USA 91, 2577–2581. 10.1073/pnas.91.7.2577 PMC43412 8146157

[CIT0008] Karchi H Miron D Benyaacov S Galili G 1995 The lysine-dependent stimulation of lysine catabolism in tobacco seed requires calcium and protein-phosphorylation. The Plant Cell 7, 1963–1970. 8535146 10.1105/tpc.7.11.1963 PMC161054

[CIT0009] Keeler SJ Maloney CL Webber PY Patterson C Hirata LT Falco SC Rice JA 1997 Expression of de novo high-lysine alpha-helical coiled-coil proteins may significantly increase the accumulated levels of lysine in mature seeds of transgenic tobacco plants. Plant Molecular Biology 34, 15–29. 9177309 10.1023/a:1005809900758

[CIT0010] Kusano M Yang Z Okazaki Y Nakabayashi R Fukushima A Saito K 2015 Using metabolomic approaches to explore chemical diversity in rice. Molecular Plant 8, 58–67. 25578272 10.1016/j.molp.2014.11.010

[CIT0011] Long X Liu Q Chan M Wang Q Sun SSM 2013 Metabolic engineering and profiling of rice with increased lysine. Plant Biotechnology Journal 11, 490–501. 23279104 10.1111/pbi.12037

[CIT0012] Shaul O Galili G 1993 Concerted regulation of lysine and threonine synthesis in tobacco plants expressing bacterial feedback-insensitive aspartate kinase and dihydrodipicolinate synthase. Plant Molecular Biology 23, 759–768. 8251629 10.1007/BF00021531

[CIT0013] Mertz ET Bates LS Nelson OE 1964 Mutant gene that changes protein composition and increases lysine content of maize endosperm. Science 17, 279–280. 10.1126/science.145.3629.279 14171571

[CIT0014] Tzchori IBT Perl A Galili G 1996 Lysine and threonine metabolism are subject to complex patterns of regulation in Arabidopsis. Plant Molecular Biology 32, 727–734. 8980524 10.1007/BF00020213

[CIT0015] Wong HW Liu Q Sun SSM 2015 Biofortification of rice with lysine using endogenous histones. Plant Molecular Biology 87, 235–248. 25512028 10.1007/s11103-014-0272-z PMC4302240

[CIT0016] Yang QQ Zhang CQ Chan MI 2016 Biofortification of rice with the essential amino acid lysine: molecular characterization, nutritional evaluation, and field performance. Journal of Experimental Botany 67, 4258–4296. 10.1093/jxb/erw209 PMC5301931 27252467

[CIT0017] Zhu XH Galili G 2003 Increased lysine synthesis coupled with a knockout of its catabolism synergistically boosts lysine content and also transregulates the metabolism of other amino acids in Arabidopsis seeds. The Plant Cell 15, 845–853. 12671081 10.1105/tpc.009647 PMC152333

[CIT0018] Zhu XH Galili G 2004 Lysine metabolism is concurrently regulated by synthesis and catabolism in both reproductive and vegetative tissues. Plant Physiology 135, 129–136. 15122025 10.1104/pp.103.037168 PMC429340

